# Incomplete isolation across a major life-history divide: genomic and geographic clines at an oviparous–viviparous lizard hybrid zone

**DOI:** 10.1098/rspb.2026.1433

**Published:** 2026-11-11

**Authors:** Darren Hunter, Maria Capstick, Oscar E. Gaggiotti, Kathryn R. Elmer

**Affiliations:** ^1^ School of Biodiversity, One Health and Veterinary Medicine, University of Glasgow, UK; ^2^ Centre for Biological Diversity, University of St Andrews, St Andrews, Fife, UK

**Keywords:** parity mode, squamate, population genomics, admixture, ancestry, cline

## Abstract

Viviparity (live-bearing) compared with oviparity (egg-laying) is among the most fundamentally distinct life-history traits seen in animals. Hybridization between different parity modes is exceedingly rare but expected to involve extensive incompatibilities. To test this, we evaluated genome-wide variation within and among individuals in a hybrid zone between oviparous and viviparous common lizards (*Zootoca vivipara*) (266 individuals; 508 448 autosomal single nucleotide polymorphisms). We found admixture between parity modes that spanned the full continuum of ancestry proportions. We identified hybrids from oviparous–viviparous crosses (i.e. F1s) and backcrosses to each parity mode, but hybrid-by-hybrid crosses (i.e. F2s) are virtually absent. Geographic clines in allele frequency have a centre within the hybrid zone, but extend considerably beyond it, reflecting broader introgression at some loci. Genomic cline analyses reveal that a small subset of loci exhibit markedly restricted introgression, with 9.7% of the 3506 ancestry-informative loci (AIM) having steep clines, indicating differential selection or genetic incompatibilities. Steep cline loci are distributed across all autosomes (full dataset; AIM: 16/17 autosomes) and have lower recombination rates. Our findings suggest that hybridization between these parity modes is long-standing; incomplete reproductive isolating mechanisms enable limited gene flow into both parental lineages, but pre- or postzygotic barriers restrict deeper hybridization.

## 1. Introduction

Hybrid zones offer a unique opportunity to study similarities and differences in the genetic architecture between two parental lineages. This is particularly true in cases where the parental lineages exhibit extreme trait differences, because novel gene combinations, resulting phenotypes and success in the environment can be inferred from genetic and demographic parameters [1–3]. The extent of reproductive isolation between lineages is a crucial determinant of the introgression dynamics at a hybrid zone. When isolation is high and there is strong selection against hybrids, the result is a sharp hybrid cline or a tension zone [4,5] that can persist for a long time with little to no introgression in either parental lineage [6]. At the other end of the spectrum is the case where hybrids have a fitness benefit over the parents; in these circumstances, a hybrid zone can quickly dissolve into a hybrid swarm and potentially a new hybrid species. Between these two extremes are a diverse range of scenarios for hybrid zone dynamics, their effects on speciation, and the manifestation of isolation through incompatibilities or divergence across the genomes [7,8].

A key component that determines the dynamics of a hybrid zone is the extent of barriers to gene flow and the abundance and location of genomic incompatibilities. Clines in allele frequency across the landscape and of excess ancestries relative to background within individuals reflect the rate and pattern of introgression across the genome during hybridization [9–11]. Clines that are steeper than the background admixture gradient can be interpreted as responding to selection across the transition, while gene flow dampens that steepness [8]. Features of a genome also play a role in the patterns of genomic introgression. For example, the extent of linkage and the rate of recombination directly influence the impact of selection on an allele in that region, and this relationship is important in speciation more generally [12,13].

Genetic cline studies have traditionally distinguished between two mechanisms that can act as barriers to gene flow between lineages: (i) intrinsic barriers affecting viability and fecundity independently of ecological context, and (ii) extrinsic barriers manifested as decreased hybrid fitness driven by environmental conditions. However, this distinction can be blurry because apparent intrinsic incompatibility may be driven by exogenous environmental factors [14]. Indeed, phenotypic differences between lineages involving fundamental life history traits can have a major impact on the ecology and evolution of species, from micro to macro scales [15–18]. A compelling example of such a phenotypic trait is parity mode. While viviparity has evolved from oviparity many times independently in amniotes and resulted in genetically divergent lineages [15,19,20], the process by which this major evolutionary transition occurs is not well understood and has never been observed in the process of change nor experimentally reconstructed [16,19,21]. Given that viviparous life histories evolved from oviparous ones with historical opportunities for mixing, it is expected—but has not been demonstrated—that selection against hybrids is strong, and there may be a reduction in fitness for intermediate phenotypes between oviparity and viviparity [22]. The rationale for this expectation is based on several points and is indirect. The widespread absence of oviparous–viviparous intermediates and/or hybrids in nature suggests poor viability, which may be for a range of physiological and genetic reasons [22]. For example, from a physiological perspective, intermediate phenotypes, such as lengthy retention of thick-shelled eggs within the mother or premature parturition of viviparous offspring, are expected to have poor viability owing to biological constraints on respiration and development [17,22]. The evolutionary divergence between oviparous and viviparous sister lineages suggests that intrinsic barriers inherent to the crossing of two genetically distinct and diverged genomes, as in Bateson–Dobzhansky–Muller incompatibilities (cf. [23]), will be significant. In addition to such neutral divergence, long-term response to historical selection for parity mode traits is reflected across the genome [16,17,24]. Thus, ecological, physiological and reproductive causes of poor fitness are expected to play a major role in selection against hybrids. These extrinsic components interact with—and arguably cannot be separated from—intrinsic genetic incompatibilities [14].

An extreme example of a hybrid zone between two lineages with very different life-history traits is found in the Eurasian common lizard (*Zootoca vivipara*). The oviparous and viviparous lineages of the species have separate biogeographic distributions, patterns of dispersal (that are expected to be genetically based) and reproductive behavioural ecologies [25–28]. In southern Austria, evolutionarily distinct oviparous and viviparous lineages hybridize despite there being a substantial divergence of approximately 5 million years between the lineages [24,29–32]. As far as we are aware, this is the only hybrid zone between an oviparous and a viviparous amniote that has been identified and characterized [17,24,33]. This population consequently is expected to reflect a component of the viability of oviparous–viviparous hybrids both in past and current conditions, even recapitulating aspects of the original divergence between parity modes [34].

This natural system presents a unique opportunity to quantify and characterize the genomic incompatibilities between oviparous and viviparous life histories. Vertebrates in general are underrepresented in the literature on genomic incompatibilities in hybrids [35], and our study provides key genome-wide and high-resolution evidence from alternative reproductive life histories. Given the notable life history and evolutionary divergence between the oviparous and viviparous lizards [24,29,36], we predict that pronounced genomic regions of high differentiation would be identified along this contact zone, with impediments to gene flow evident across the genome. We test these hypotheses using low- to moderate-coverage whole-genome sequencing of pure oviparous, pure viviparous and a range of hybrid individuals at high resolution. Using analyses of genomic and demographic population structure, we infer the pattern and extent of genomic admixture between parity modes across all autosomes. From a demographic perspective, we examine the abundance of first-generation and backcross hybrids in the population to consider the reproductive patterns driving ancestry breakdown in this population. We evaluate both the geographic clines and the genomic clines to map the scale and strength of selection across the genome in the transition between parity modes. We sampled a wild population of adult individuals, and therefore gene flow and admixture reflect both ecological and genomic sources of reproductive isolation [14,23]. Our findings demonstrate how complex, genome-wide histories of hybridization and selection manifest at very fine spatial scales across the landscape.

## 2. Methods

More detailed methods can be found in the supplementary material.

### (a) Biological sampling

We whole-genome sequenced 162 females and 38 males from previous collections [24,37,38] across the study location (supplementary material, figure S1). Briefly, whole genome libraries were prepared and then sequenced on Illumina NovaSeq platform (target: 9 Gbp/individual). New sequence data were combined with previously generated data for an additional 66 individuals (NCBI SRP279965) for a total *n* = 266 (coverage from 2.41 to 16.04×; supplementary material, table S1). Reads were aligned to a draft genome assembly of the Eastern Oviparous lineage (Eovi.ref.V1.19Chr) [29], which is 1.40 Gb consisting of 17 autosomes and Z and W sex chromosomes. Only the autosomal regions are analysed here, because: i) there are suspected differences in the sex-chromosome system between the lineages (Z1Z2W vs Zw), ii) hemizygosity makes autosomes and allosomes not strictly comparable (and therefore males and females in our study), and iii) sex chromosomes have little or no recombination and our focus is on admixture between lineages. SNPs were called using bcftools v1.16 [39] retaining genotype likelihoods. After filtering, the mean per sample coverage was 6.95x (range: 2.41-16.04x). Beagle v5.4 [40] was used to phase the data and impute missing genotypes. This imputed dataset was only used when software was unable to handle missing data; unless stated otherwise the non-imputed version was used. Prior to linkage disequilibrium pruning the dataset consisted of 13 069 871 SNPs across the 17 autosomes (Z and W sex chromosomes were excluded) for 266 individuals. After LD pruning the SNP number reduced to 508 448.

### (b) Population genomic analyses

Plink was used to calculate Wright's FST values and the PCA. ADMIXTURE [41] was used to estimate individual ancestry proportions using the LD-pruned SNP data set and 1000 bootstrap replicates. Individuals with an ancestry proportion of >95% or <5% were assigned to oviparous/viviparous lineage and individuals with *q*-values 0.05–0.95 were classified as hybrids. The hierfstat package v0.5.11 [42] was used to calculate summary statistics. The q and Q12 values (±95% HPD) were calculated in entropy v2.0 [43] from ~10% of the variants (50 734 autosomal SNPs) and individuals were classified as F1 hybrids, F2 hybrids (offspring from two F1s), parental backcrosses (hybrids into pure lineage crosses), or parental lineages (pure oviparous or viviparous) based on the HPD interval overlapping the q and Q12 expectations for each category.

### (c) Genomic and geographic cline analyses

For geographic cline analysis, a 1-dimensional transect was approximated from the 2-dimensional sampling scheme (supplementary material, figure S1). Ancestrally informative markers (3506 loci; those with FST of 0.7 or higher between parental lineages) were subset from the imputed genotypes. Individuals were grouped into 50 m intervals and the average allele frequency for each SNP in each group was calculated. Geographic and genomic clines analyses were carried out using the bgchm R package v0.0.0.9000 [10,44]. Outliers were determined based on the 90% equal-tail probability intervals excluding 1 for the cline gradient, or 0.5 for the cline centre. Cline gradients < 1 are classified as shallow clines, while gradients > 1 are considered steep clines [10,44]. Loci with cline centres below 0.5 are considered to have a credible excess of oviparous ancestry, while cline centre above 0.5 suggests a credible excess of viviparous ancestry. Genomic cline analysis was additionally carried out for all 508 448 loci.

Heterozygosity, FIS, linkage disequilibrium, and admixture value modality along the transect were calculated for the full SNP dataset, with individuals split into 10 groups.

### (d) Local recombination rate inference

Autosomal markers were lifted over from a linkage map placed on a *Z. vivipara* reference genome [45] from a different lineage to the Eastern Oviparous lineage genome. Chromosomes 9 and 11 were excluded as they lifted over very poorly. In total, 15 087 autosomal SNPs were successfully lifted over and 14 899 were mapped onto the expected chromosome. Splines were fitted to 25 separate regions containing between 54 and 1215 loci. To evaluate genomic cline steepness and recombination rates, simple linear regressions were conducted on the SNP-specific values and the chromosome averages [46].

**Figure 1.**
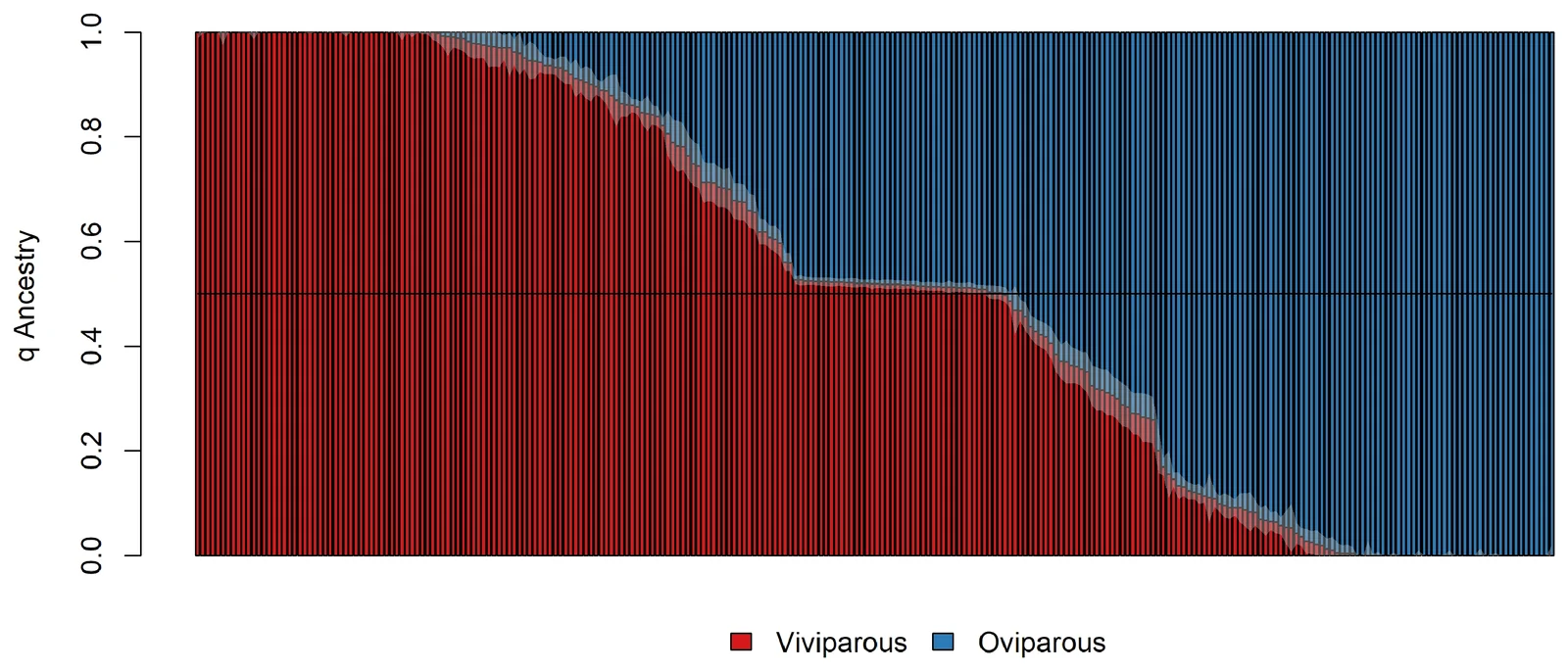
Genome wide ancestry for each individual estimated using ADMIXTURE, showing the proportion of viviparous ancestry (red) and oviparous ancestry (blue). Certainty in each individual estimate is plotted in white, using twice the s.e.m. calculated from 1000 bootstraps. An ancestry *q*-value of 0.5 is indicated by the horizontal line.

## 3. Results

### (a) Population variation at the hybrid zone

The 266 lizards were collected from across the hybrid zone area, spanning approximately 1.07 by 0.91 km and including a change in elevation from 1357 to 1750 m (supplementary material, figures S1 and S2). All levels of admixture proportions were present in individuals across the population. Fifty-one individuals were pure oviparous (found at lower elevations) and 60 were pure viviparous (found at higher elevations) (where pure is a *q*-value of 95% or greater for either parity mode lineage; referred to simply as ‘oviparous’ or ‘viviparous’). The s.e.m. from bootstrapping indicated that admixture values were assigned with high certainty, especially at values of 0, 1 and 0.5 (figure 1), and therefore we focus on the point estimates. A total of 46 individuals (i.e. 23% of sampled individuals from this population) were fully intermediate hybrids, meaning they had *q*-values between 0.45 and 0.55.

PCA analyses showed a separation of individuals representing viviparous and oviparous genomes, with parity mode explaining a substantial proportion of variation in the dataset (PC1 = 48.7%; PC2 = 4.87%) (supplementary material, figure S3). The PCA is symmetrical, with hybrids in the middle and similar levels of variation explained in viviparous and oviparous individuals (along PC1). The variation along PC2 is somewhat greater for oviparous than viviparous groupings.

The level of population genetic diversity was reflective of large population sizes and high diversity. Observed heterozygosity was 0.54 for the oviparous population, 0.56 for the viviparous population and within hybrids was elevated at 0.681. Within-population gene diversity was 0.364 for the oviparous population, 0.369 for the viviparous population and 0.421 in the hybrid population.

Along the transect, *F*_IS_ values were lowest at the extremes and elevated in the middle spatial area, consistent with inter-lineage interbreeding within the transect (supplementary material, figure S4a). However, the confidence intervals are all overlapping, and the signal in the dataset is subtle. Linkage disequilibrium (*r*^2^) between neighbouring SNPs was relatively consistent across the transect (supplementary material, figure S4b). Visualization of the admixture proportions (*q*-values) along the spatial transect showed the modality shift from higher proportions of pure lineages at either end or intermediate admixture proportions in the middle zones (supplementary material, figure S4c).

An analysis of *F*_ST_ across the genome of all individuals identified relatively few lineage-specific SNPs: on the autosomes there were 330 (0.065%) SNPs with an *F*_ST_ greater than 0.95, of which 107 (0.021%) SNPs were found to be completely lineage-specific (i.e. *F*_ST_ of 1 between oviparous and viviparous, allowing 0.05 for potential genotyping errors) (supplementary material, figure S5). Average *F*_ST_ between the oviparous and viviparous lineages across all autosomes was 0.113. Additionally, 40623 (7.99%) of autosomal variants are monomorphic only in the viviparous individuals and 47 238 (9.29%) are monomorphic in oviparous individuals (supplementary material, table S2).

### (b) Hybrid population is variable and backcrossed

Analysis of ancestry proportions to infer the genetic history of individuals showed that most hybrids are the result of crosses with pure oviparous or viviparous individuals rather than among hybrids. Only a single individual showed evidence of both parents being substantively introgressed (*Q*12 = 0.689, *q* = 0.454), while all other *Q*12- and *q*-value associations were indicative of at least one parent being oviparous or viviparous (figure 2). Based on each individual's 95% HPD encompassing the expected *q* and *Q*12 values of a given hybrid category, a total of 39 individuals were classified as F1 (*Q*12 = 1, *q* = 0.5), 55 individuals were classified as oviparous and 70 individuals were classified as viviparous (*Q*12 = 0, *q* = 0 or 1) (supplementary material, table S3). At more complex levels of introgression, only two individuals were classified as first-generation hybrid-to-oviparous backcrosses (*Q12* = 0.5, *q* = 0.25) and a single individual was classified as a first-generation hybrid-to-viviparous backcross (*Q*12 = 0.5, *q* = 0.75). The remaining individuals have genetic ancestry signatures suggestive of deeper backcrosses (0 < *Q*12 < 1 and *Q*12 ≠ 0.5) between partial hybrids and pure viviparous individuals (46 individuals) and pure oviparous individuals (53 individuals) (supplementary material, table S3). An exact binomial test indicates similar proportions of deep viviparous and oviparous backcrossed individuals in the population (46 successes out of 99 trials, expected proportion 0.5, *p-*value = 0.547).

**Figure 2.**
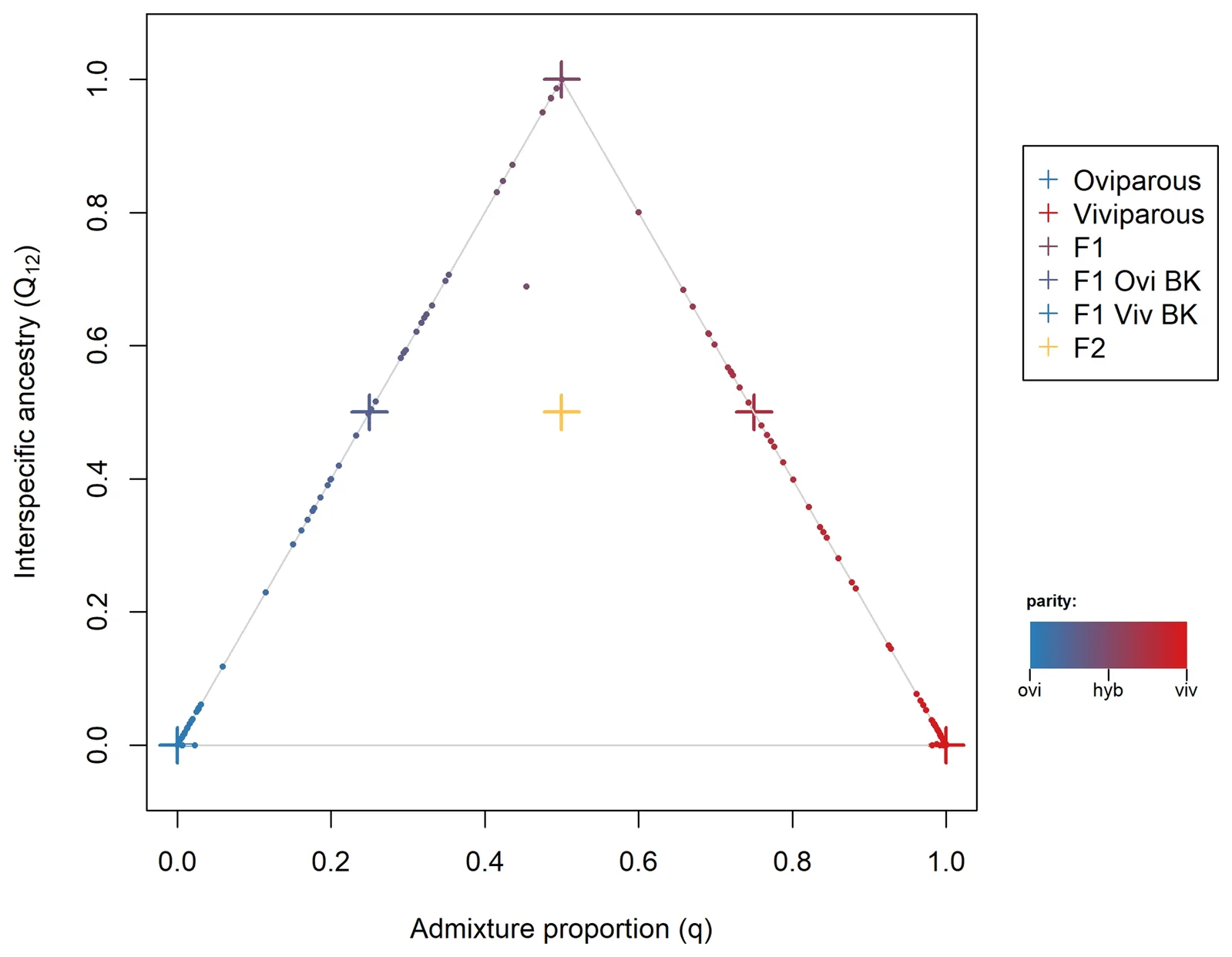
Plotted admixture proportions (*q*) and interspecific ancestry (*Q*12) values as inferred by ENTROPY analysis. The cross symbols show the theoretical location of early hybrids (i.e. F1s and F2s) and backcrosses relative to parental lineages. The solid line shows the expectation when one parent is from a parental lineage. Analysis is based on 50 734 autosomal SNPs (*N* = 266).

### (c) Geographic clines at ancestry-informative loci

From the spatial distribution of oviparous, viviparous and hybrid individuals on the landscape and the geographic clines at the 3506 AIM, it is evident that the transition between oviparous and viviparous allele frequencies occurs quickly (figure 3). The cline centre estimates ranged from –2.00 to 2.29 km (figure 3), after excluding 21 SNPs with very shallow clines (these generated extreme values of –232.8 to 9969.4 km and are presumed to be artefacts). The median cline centre lies robustly within the sample transect. The cline widths using the median of the posterior distribution ranged from 0.197 to 20.09 km (figure 3). Geographic cline centres and cline widths increase together, expanding both positively and negatively from the cline centre, such that clines shifted outside the centre of the transect tended to have larger widths. A regression analysis showed there is a very slight positive relationship between cline centres and cline widths (slope = 0.019, SE = 0.002, *p* < 0.001; supplementary material, figure S6), but the effect is very small. For those SNPs in the model that are fixed between the two lineages (107 loci), the median of the predicted cline centres is 0.26 km, i.e. in the lower spatial zone of the transect and the median of the predicted cline widths is 3.08 km (supplementary material, figure S7). Overall, the geographic clines analysis indicates a genetic transition between the two lineages within the hybrid zone sampled and that extends beyond our scale of sampling, which is reflected in substantial cline widths and extensive introgression.

**Figure 3.**
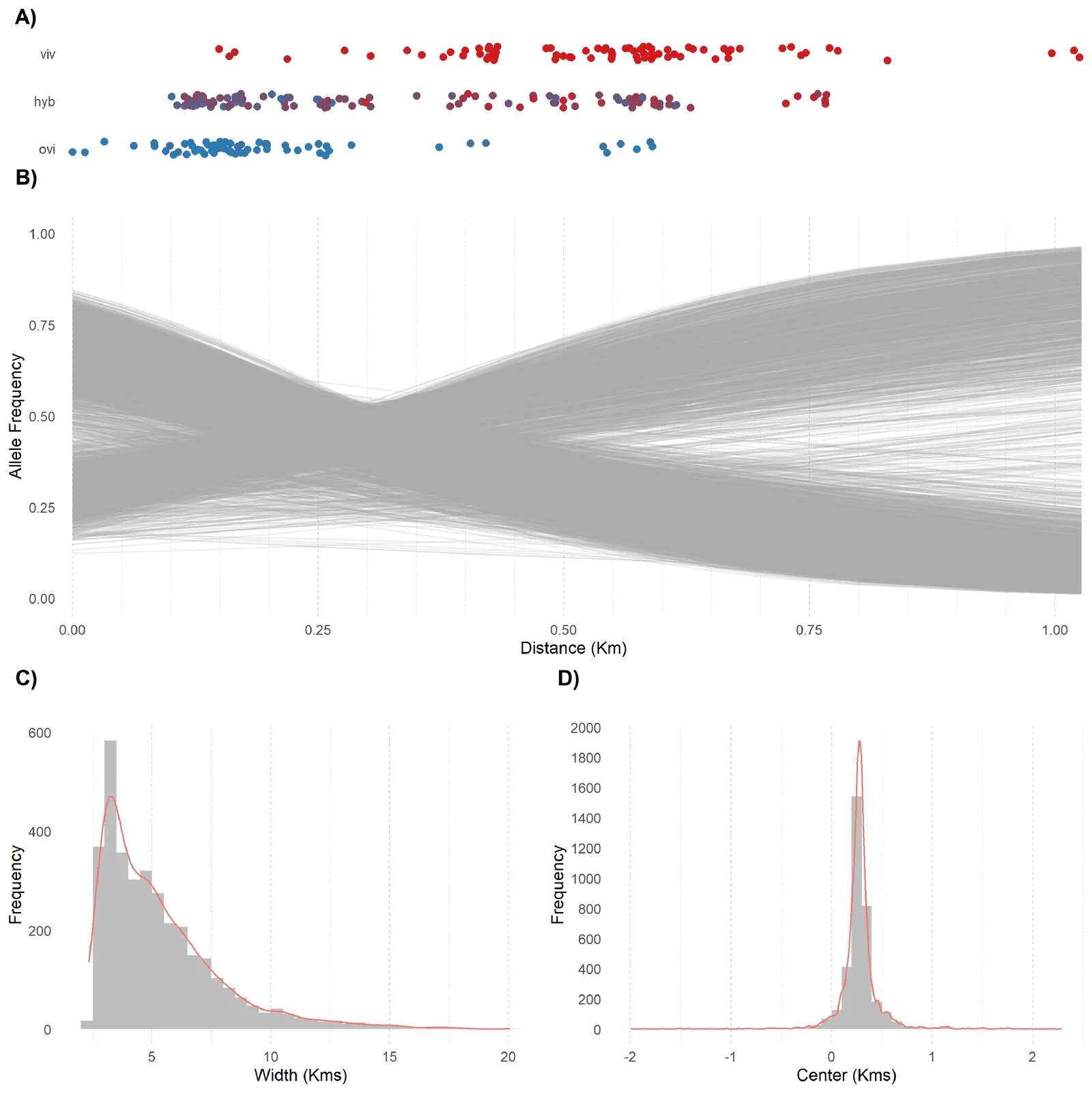
Geographic clines, along the linear transect of the hybrid zone, of the 3506 AIMs. SNPs plotted on the logit scale, inferred with bgc-hm. The coloured points in panel (A) represent the location on the transect of sampled individuals, split into oviparous, hybrid and viviparous individuals and coloured from blue to red, based on admixture *q*-values. (B) The change in allele frequency across the transect. (C) Geographic cline widths and (D) cline centres of the AIM (with 21 outliers removed) between lineages, along with a smoothed approximation of the distribution shape, in red.

### (d) Genomic clines at ancestry-informative loci

Across the 3506 AIMs, there was significant variation in the patterns of introgression (figure 4A). Relative to genome-average ancestry, a total of 926 loci deviated from the null expectation, including 68 loci that were outliers for both cline slope and cline centre. Of these, 342 had a credibly steep cline and 312 had credibly shallow clines (supplementary material, table S5). Regarding cline centres, 195 loci had values indicating excess oviparous ancestry while 145 loci had centres indicating excess viviparous ancestry. Most loci having credibly shallow genomic clines were neutral for excess ancestry (302 out of 312 loci), suggesting that when barriers to gene flow are low, then introgression acts evenly in both directions. Most credibly steep cline loci were also neutral for excess ancestry (284 out of 382 loci). However, of those loci in a steep cline, 12% (41 out of 342 loci) display excess oviparous ancestry, suggesting introgression is primarily from the oviparous lineage, and alleles from this lineage may confer a selective advantage, while 5% (17 out of 342) display excess viviparous ancestry. All four types of outlier loci were distributed across the genome and were found on every autosome (figure 4B; supplementary material, tables S4-S6). Genomic cline analysis of all 508 448 loci follows an overall similar pattern (supplementary material, tables S7 and S8).

**Figure 4.**
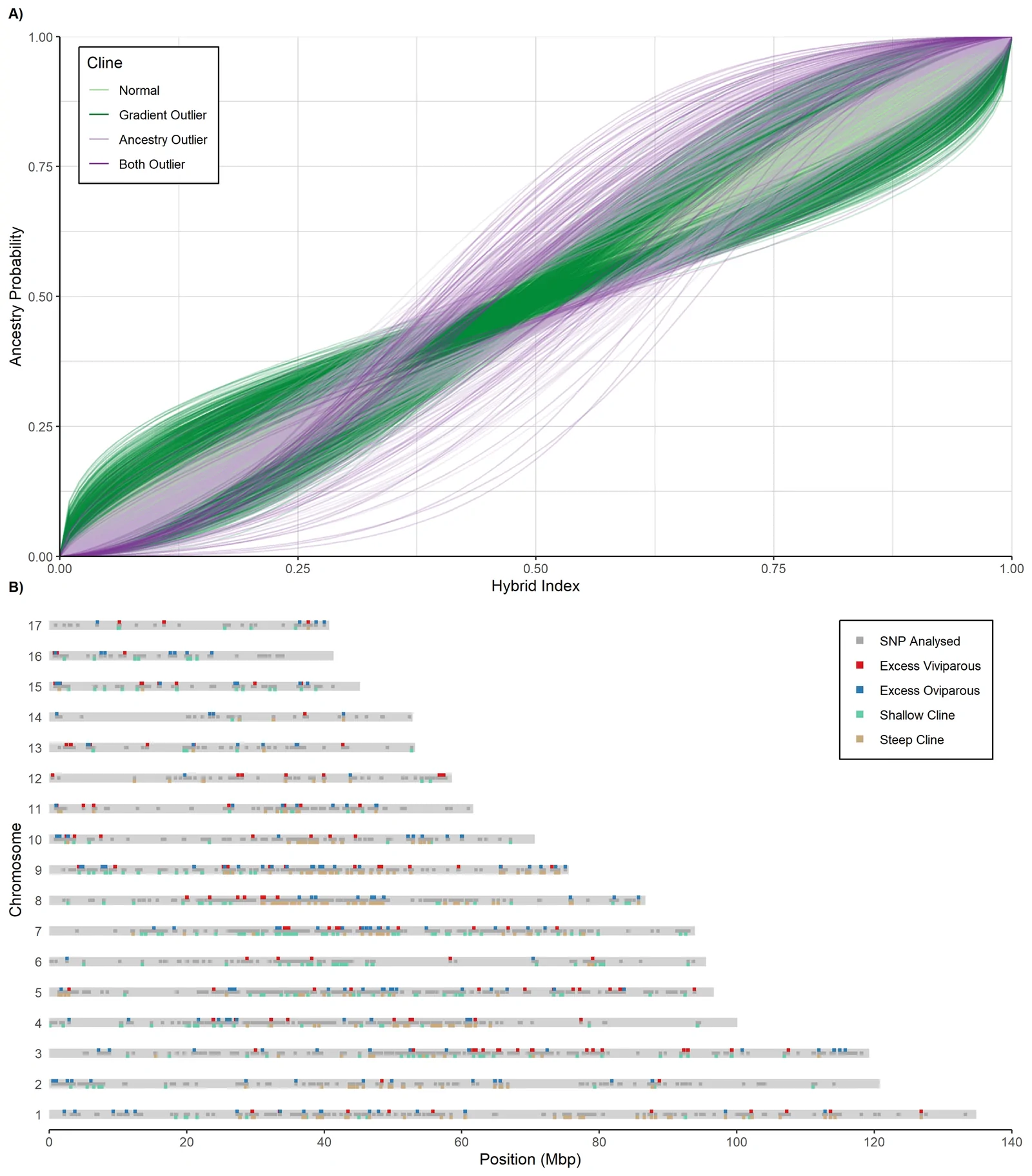
(A) Genomic clines for the 3506 AIMs. Gradient outliers are presented in dark green, centre outliers in light purple and outliers of both in dark purple. (B) Location in the genome of the 3506 AIMs with cline centre [excess ancestry] outliers highlighted above the lines and cline width outliers highlighted below the lines.

### (e) Recombination and excess regions

SNP-specific recombination rates ranged from 0 to 25.02 cM Mb^–1^. The extremes of these predictions are likely to be a consequence of imperfect lifting-over rather than biologically meaningful, but general patterns are still captured. The average recombination rate varied from 1.256 cM Mb^–1^ on chromosome 4 to 2.457 cM Mb^–1^ on chromosome 17 (supplementary material, table S9). At the SNP level, steeper genomic clines show slightly lower recombination rates (slope = –0.004, *p-*value < 0.001). This relationship between average genomic cline slope and average recombination rate per chromosome is negative; when considering whole chromosomes, lower recombination rates are associated with stronger genomic clines (slope = –0.013, *p-*value = 0.014; supplementary material, figure S8).

## 4. Discussion

We found that there is admixture across the autosomal genome between oviparous and viviparous lizards in this hybrid zone. This contrasts with our predictions, as we expected many substantial regions of divergence within the genome, which would reflect extensive incompatibilities and barriers to admixture between these life histories. Evidence of selection against some hybrid genotypes was observed in loci showing steep-slope genomic clines relative to the background admixture gradient, and these loci were widely distributed across the genome. The transition between pure oviparous and viviparous individuals is sharp across the landscape, with their overlap spanning approximately 450 m. This abruptness likely reflects the rarity of hybrids and the absence of later-generation hybrids, consistent with strong reproductive isolation. Ancestry proportions show that low, symmetric backcrossing is occurring in the direction of both lineages, but there are few hybrid-by-hybrid crosses. Reproductive isolation owing to genomic incompatibilities, poor fitness of hybrids for genetic or ecological reasons, sexual selection or demographic effects might all contribute to this outcome.

### (a) Hybrid ancestry past and present

In admixture zones, the present-day genetic composition of individuals is the result of both past and contemporary hybridization [47]. We found the proportion of admixture across autosomes spanned the full range of possible oviparous–viviparous contributions and with a high level of symmetry into both genomic backgrounds (figure 1). Such genome-wide symmetrical introgression is rare; for example, in other squamates, such as wall lizards [48] or rattlesnakes [49], introgression is often asymmetric owing to selection and/or genomic features. Our dense geographical and genomic sampling enabled detection of the full extant range of genomic combinations. This symmetry reflects a striking lack of genome-wide barriers to gene flow and incomplete reproductive isolation, allowing crosses (though at low frequencies) back into both ancestries.

Our demographic analysis showed that the intermediate genotypes (*q* approx. 0.5) are indeed F1 hybrids (figure 2), from pure viviparous by oviparous mating [43]. Previous research on common lizards has shown that such genetically admixed hybrid females also have intermediate reproductive traits between typical oviparity and viviparity, including thin eggshells and intermediate gestation and developmental stages [24]. While there is evidence that offspring from hybrid females are to some extent viable, these traits between oviparous and viviparous reproduction are expected to have a fitness cost [17,22,24]. These potential costs have important implications for early stages of the evolution of viviparity, where incomplete reproductive isolation and intermediate phenotypes would be expected [17].

The distribution of genomic ancestries indicates there is backcrossing from hybrid individuals into pure lineages at similar levels and towards both parity modes. However, F1 backcrosses and pure hybrid-by-pure hybrid crosses are rare. Only two individuals were from F1 hybrid-to-oviparous backcrosses, one F1 hybrid-to-viviparous backcross, and one individual was a likely F2 (i.e. F1 × F1 hybrid cross). This emphasizes the paucity of hybrid reproduction, consistent with the global rarity of oviparous-viviparous hybridization in squamates [17]. Our findings from a natural population mirror those from lab crosses in common lizards, which suggested few behavioural isolating mechanisms but some postzygotic isolation [50].

Several explanations exist for the absence of later-generation hybrids, and these have different ecological and evolutionary consequences for hybrid fitness and population trajectories [14]. Genetic or intrinsic selection against hybrid–hybrid matings (e.g. hybrid breakdown) may limit F2 survival [23]. Alternatively, sex-specific selection or sex-specific incompatibilities may restrict successful reproduction to certain crosses [14,23]. Sexual selection against F1 hybrids has wide-ranging impacts on population evolution, from genomic to behavioural consequences of reproduction, and these consequences are not siloed [14,51]. Natural and sexual selection also interact, as found in hybrid flycatchers where the consequences for sexual selection and genetic incompatibilities differ for male versus female hybrids [52]. The life stage and strength of selection against hybrids remain unclear but are key to understanding fitness consequences.

The dearth of later-generation hybrids may also simply reflect the low frequency of F1 individuals in the population, limiting opportunities for mating and facilitating backcrossing with pure oviparous or viviparous individuals [35]. Given the species' low dispersal [27,28], the few alternative backcross hybrids, and the important phenotypic consequences of hybridization (at least for females) [24], we consider this explanation to be less likely, but one that certainly warrants further investigation [17,29,53].

Ultimately, demographic factors, genomic incompatibilities and reproductive isolation are not mutually exclusive in their contribution to the extant patterns of hybridization [9,23]. Future studies should aim to further disentangle their impacts.

### (b) Heterogeneous barriers between parity modes

In hybrid zones, gene flow can persist while small genomic regions maintain divergence [54,55]. Intrinsic aspects of genomes, such as structural features and recombination, further influence the distribution and extent of ancestry tracts [9,56]. Consistent with this, the combined signal from our geographic and genomic cline analyses indicates that this hybrid zone is maintained by strong reproductive isolation—likely intrinsic postzygotic selection—resulting in narrow and largely coincident allele frequency transitions (figure 3). While introgression occurs widely across the genome, a subset of loci exhibit steep genomic clines (figure 4) characteristic of barrier regions under strong selection [1,9]. The excess oviparous ancestry at a fraction of these loci (12% versus 5% viviparous) suggests asymmetric selection favouring oviparous alleles [47].

Recombination rate tended to be lower at loci with steep genomic clines. Chromosomes with steeper cline slopes also showed lower recombination (supplementary material, figure S8). This inverse relationship is expected in hybrid zones with extensive genetic mixing, when selection against introgressed alleles can affect larger, linked genomic regions and reduce gene flow, resulting in steeper clines [57]. Linkage disequilibrium showed no clear pattern across the transect (supplementary material, Figure S3), but we caution that the resolution of this analysis will be low. Overall, low linkage disequilibrium and widespread admixture suggest long-term hybridization [58], consistent with species-wide inference [29]. By contrast, research on hybrid rattlesnakes found that regions of excess ancestry had either no difference or elevated recombination rates, depending upon the species recombination map used [49]. Whether migration increases or decreases the junctions of, or the extent of, ancestry tracts is influenced by gene flow and population size [58,59]. Enrichment of steep clines in low recombination autosomal regions further supports a role for linked selection and coupling among barrier loci [49,56]. The genomic landscape of recombination remains to be more finely characterized within lineages for common lizards.

Two limitations apply to our genomic analyses. First, the spatial scale of sampling was restricted: at both higher and lower elevations, the habitat changes and lizards are less abundant. This restricted design may systematically underestimate cline steepness and directional introgression, causing us to be conservative in our inference of the strength and extent of barriers to gene flow [1,48]. Specifically, the inference of ‘pure’ parental allele frequencies may be biased relative to the rest of the distributions of these lineages, where introgression is effectively absent [29]. Future research would benefit from sampling outside the hybrid zone. Second, we focused specifically on autosomes because sex chromosomes are complex and variable in this species [36] and currently not well characterized. The females are heterogametic, and there have been fusions and translocations of the sex chromosomes and these differ among lineages [36]. It is possible that sex chromosomes play a role in reproductive isolation, as they do in wall lizards [48], and may show substantially different patterns of introgression than do autosomes. This is a fruitful area for future research.

### (c) Hybrids on the landscape

Hybridization patterns reflect both genetic and ecological processes. While we have not characterized environmental features, in general these two lineages occupy similar habitat [27,60,61] and at this spatial scale there is little or no detectable environmental difference other than the small (approx. 50 m) increase in elevation. Thus, patterns we find are unlike other lizards, such as *Sceloporus woodi* and *S. undulatus undulatus*, where hybrids occur between distinct habitat patches, suggestive of ecological selection [62]. At broader biogeographic scales, the oviparous lineage has a more restricted distribution at lower elevations [63] and limited post-glacial expansion [30], whereas the viviparous lineage has a broader environmental tolerance, inhabits higher elevations [63], and tends to have greater dispersal distance [27]. This expansive geographic distribution is consistent with the selective advantage of viviparity in challenging climates, facilitating female control over gestation and resilience to environmental threats [64,65].

We suggest that environmentally dependent selection maintains the hybrid zone, given the narrow distribution of geographic cline centre values and symmetric introgression [4,9,66]. While our overall sampling was extensive, subgroup sizes along the transect are smaller and results should be interpreted cautiously. Slightly elevated heterozygosity occurred in the centre, where hybrids are most abundant. The pattern we uncovered is somewhat in contrast to wall lizards, where numerous intrinsic barriers and less symmetric admixture were found [48]. Our high-resolution sampling reduces bias from missing data and accurately captures population structure. The use of simulations to explore potential scenarios responsible for patterns we identified will be an important next step to quantifying these effects.

### (d) Hybridization and the genetic origins of viviparity

Our result is somewhat counterintuitive: because oviparous viviparous hybrid zones are so rare (effectively absent) in nature [17], it is surprising that such a range of introgressed backgrounds exist at this site. As far as we are aware, this is the only recorded common lizard hybrid zone [33,67,68] and, more dramatically, the only hybridization between an oviparous and a viviparous amniote [15,17,24]. Why such hybrids are so exceedingly rare among sister lineages differing in parity mode—given that they likely existed at early stages of viviparity evolution in all vertebrates—is an open question [17,69,70].

Despite extensive introgression, a small number of loci may strongly influence reproductive barriers. Previous research on the genetic basis of reproductive traits found that *Z. vivipara* chromosome 8 (named chromosome 7 or ChrLG7 in [24]) was enriched for phenotypic associations and functional loci [24]. Specifically, gestation time and eggshell traits were enriched, including SNPs highly associated with the mammalian pregnancy gene LYPLA1 [24]. We also found loci with fixed *F*_ST_ between lineages in that same chromosome (particularly between 31 and 42 Mbp on Chr 8). However, it is not the chromosome having the highest proportion or absolute number of steep cline loci, nor having the lowest recombination rate in cM Mb^–1^. Our study focuses on introgression patterns across the population and genome for males and females, without considering parity traits. Recombination and admixture are occurring across the autosomes, but some regions for female reproduction may be playing a major role in maintaining the distinctiveness between parity mode lineages [2,4,9,24]. Genome-wide admixture but extremely limited extent of hybridization suggests that few genomic regions of large effect are maintaining the isolation of oviparous and viviparous lineages. Studies to measure fitness and phenotypic consequences of hybridization for both sexes and parity modes will be valuable to disentangle these effects.

Evolutionary genomic patterns occurring in modern hybrid zones are mirrored in ancient hybrid lineages [34]. In common lizards, these hybrids provide insight into the genetic conditions that might have existed at the very early stages of viviparity evolving from oviparity. Several outcomes from this research will be valuable for informing future models of selection for and against hybrids. For example, we have shown that there are no large autosomal regions that strongly resist introgression, although it is known there are many regions of the genome associated with parity mode traits, such as eggshell thickness and incubation time [24]. We show that backcrosses into both viviparous and oviparous backgrounds are viable, which reinforces how environmental context is key in the life history trade-offs of viviparity [71,72]. Using quantitative predictions generated here, evolutionary scenarios for genetic, ecological (including physiological) and sexual components of selection can be reconstructed.

## 5. Conclusion

Hybrids are immensely informative about how reproductive mode transitions occur, the genomic regions involved and the underlying pattern of differential adaptation. This hybrid zone offers rarely available empirical evidence about crosses between oviparous-viviparous life-histories. A substantial proportion of individuals in our study (23%) were intermediate hybrids, revealed by dense spatial sampling (approx. 1 lizard per 5 m). Hybridization is symmetric and introgression and admixture are seen across the genome, suggesting there are limited genomic barriers. However, genomic cline analyses show effects genome-wide, with approximately 10% of all AIM having a steep cline, indicative of strong selection for reduced introgression. The very limited spatial scale of the hybrid zone suggests there are substantial ecological and genetic consequences to hybridization. Future research should track hybrid fitness and pinpoint mechanisms limiting viability in this context. These inferences provide insight into the role of selection in the evolution of viviparity.

## Supplementary Material



## Data Availability

All raw sequence data are publicly available on NCBI (BioProject for new data: PRJNA1479429, selected sequences from PRJNA657575) and accession numbers for accessing each sample are listed in the supplementary material, table S1. Processed variant files, intermediate and database files and custom scripts are permanently available on Univ. of Glasgow Enlighten Repository http://dx.doi.org/10.5525/gla.researchdata.2312. Supplementary material is available online [73].
