# Supplementary material for: Incomplete isolation across a major life-history divide: genomic and geographic clines at an oviparous–viviparous lizard hybrid zone
Source: Proc Biol Sci. 2026 Nov 11;293(2082):20261433. doi: 10.1098/rspb.2026.1433 (PMC13750935; doi:10.1098/rspb.2026.1433)
Supplement: Supplementary file 1 [file rspb.2026.1433.zip › rspb.2026.1433/rspb20261433_si_001.pdf]

SUPPLEMENTARY MATERIAL

Incomplete isolation across a major life-history divide: genomic and geographic clines at an oviparous–viviparous lizard hybrid zone

Darren Hunter, Maria Capstick, Oscar E Gaggiotti, Kathryn R Elmer

Proceedings of the Royal Society B

Doi: 10.1098/rspb.2026.1433

Supplementary Methods:

Biological sampling

We whole genome sequenced 162 females and 38 males using samples that had been collected in previous research (*1-3*). This included presumed hybrids (60 female, 27 male) and 46 non-hybrids, based on previous genotyping or phenotyping (*1-3*). Additionally, 67 individuals were selected to be as spatially diverse a sample set as possible across the hybrid zone, based on their geographical coordinates and unbiased with respect to hybrid status. This was achieved using Weakly Associated Vectors (WAVE) sampling implemented in the WaveSampling package (*4*) in R v4.0.4 (*5*), which minimises autocorrelation by a spatial weights matrix. The individuals included in the dataset spanned 1.07 km by 0.91 km area from 1357 to 1750 m elevation (Supplementary Figure 1).

DNA was extracted using standard commercial columns (Mackary-Nagel). Whole genome libraries were prepared using NEBNext Ultra II FS DNA Library Prep kit (New England Biolabs) with reagent volume halved. In brief, 100ng DNA per individual was fragmented for 8 minutes, ligated with adaptors, and size selected using double-sided bead cleanup (New England Biolabs). Adaptor-ligated DNA was PCR-enriched using Illumina Index primers for 7 cycles, and each reaction was cleaned using NEB purification beads. Sample libraries were quantitated and a subset were run on Tapestation (Agilent) to assess size distribution. Samples in each library pool were equimolared using the subset average bp determined from the Tapestation profiles. Final library pools were cleaned, then concentration and size confirmed. Libraries were sequenced at Novogene (UK) on an Illumina NovaSeq platform to a target of 9 Gbp per individual (2x150bp).

Genome data processing, mapping, and variant calling

New sequence data were combined with previously generated data for an additional 66 individuals (65 females, 1 male; NCBI SRP279965) from (*1*) for a total sample size of 266

(Supplementary Table 1). Raw reads were quality checked using FastQC v0.11.9. Potential bacterial, viral, invertebrate, and vertebrate contaminates were screened for using FastQ Screen v0.15.1 (*6*). Only three individuals showed some evidence of contamination (>2.5% unmapped reads to reference genome or >10% multiple hits to multiple genomes for any other species) and were processed with Kraken 2 v2.1.2 (*7*) using the standard database (6/7/2022) with default settings to remove non-target sequence. Individual coverage ranged from 2.41 to 16.04x (Supplementary Table 1).

Trimmomatic v0.39 (*8*) was used in PE mode to trim TruSeq3-PE-2 Illumina adapters and to trim low quality (below 3) and N bases from both ends, retaining only reads >36 bases. Trimmed reads were aligned to a draft genome assembly of the Eastern Oviparous lineage (Eovi.ref.V1.19Chr) (*9*) using Bwa-mem2 v2.1 mem (*10*). This genome is 1.40 Gb and consists of 17 autosomes and Z and W sex chromosomes. Only the autosomal regions are analysed here, because: i) there are suspected differences in the sex-chromosome system between the lineages (Z1Z2W vs Zw), ii) hemizygosity makes autosomes and allosomes not strictly comparable (and therefore males and females in our study), and iii) sex chromosomes have little or no recombination and our focus is on admixture between lineages. Chromosome coordinates and naming are not strictly comparable to previous studies because of different reference genomes (*9*, *11*). Samtools v1.11 (*12*) was used to sort and index the bam alignment files. Duplicates were identified and marked using Picard MarkDuplicates v .24.1-2-g4a9c42d-SNAPSHOT (“Picard toolkit,” 2019). Each bam file was evaluated using QualiMap v.2.2.1 (*13*) to acquire summary statistics.

SNPs were called using the mpileup and call tools from bcftools v1.16 (*12*) using the default settings and retaining genotype likelihoods. Initial filtering was carried out to remove variants in the extreme ranges of the distribution for Mapping Quality (MQBZ), SNP Base Quality (BQBZ), Position (RPBZ) and Soft-clips (SCBZ), based on density plots of these values. Variants that fell outside the following ranges were removed using bcftools filter: 20 ≤ MQBZ ≥ 5, -7 ≤ BQBZ ≥ 7, -10 ≤ RPBZ ≥ 10, and -7 ≤ SCBZ ≥ 19. Any variants fixed across all individuals or completely missing following this filtering were also removed. Further quality filtering was carried out removing variants with a genotype read depth below 3 or greater than 40, or variants with a genotype quality score below 20. SNPs with quality score <300 were excluded as were those within 10 base pairs from an indel and any resulting monomorphic sites. The only variants retained were biallelic SNPs with a minor allele frequency greater than 0.05. Finally, sites with more than 60% missing genotypes were

removed. After filtering, the mean coverage across all 266 samples was 6.95x and ranged from 2.41x (sd = 4.50) to 16.04x (sd = 14.50x). The final vcf file was converted to binary format using Plink v1.90b7 (*14*). Linkage disequilibrium was inferred in Plink using the --indep-pairwise flag with a window size of 50 variants, a shift of 10 variants and an r2 value of 0.1.

Beagle v5.4 (*15*) without a genetic map was used to phase the data and impute missing genotypes. This imputed dataset was only used when software was unable to handle missing data; unless stated otherwise the non-imputed version was used. Prior to linkage disequilibrium pruning the final dataset consisted of 13,069,871 SNPs across the 17 autosomes (Z and W sex chromosomes were excluded) for 266 individuals. After LD pruning the SNP number reduced to 508,448.

Population Genomic Analyses

Plink was used calculate the variant-specific and overall Wright's F_{ST} values between the two lineages. A PCA analysis, using the imputed dataset, was also carried out in Plink. ADMIXTURE (*16*) was used to estimate individual ancestry proportions, assuming two populations (i.e. oviparous or viviparous), using the LD-pruned autosomal SNP set (508,448 SNPs, 266 individuals) and 1000 bootstrap replicates. Using values from ADMIXTURE, individuals with an ancestry proportion of >95% or <5% were assigned to a lineage and individuals with q-values 0.05-0.95 were classified as hybrids. The hierfstat package v0.5.11 (*17*) was used in R to calculate relative values of observed heterozygosity (Ho), within population gene diversity (Hs), and Wright’s inbreeding coefficient (F_{IS}) for the oviparous, viviparous, and hybrid subpopulations.

To establish the hybrid population composition, we used entropy v2.0 (*18*). The genotype-likelihood model was used and approximately 10% of the variants (50 734 autosomal SNPs) were randomly selected from the LD pruned dataset (N = 266). The model was repeated three times, each with a burn-in of 20,000 followed by 10,000 iterations and thinning every 10 samples, resulting in a posterior sample size of 1000. The mean admixture proportion (q) and ancestry complement from each parental lineage (Q12) were checked visually to ensure convergence and mixing, and then the runs were combined. The q and Q12 values, along with the 95% HPD intervals, were then calculated for each individual. Individuals were classified as F1 hybrids, F2 hybrids (offspring from two F1s), parental backcrosses (hybrids into pure lineage crosses), or parental lineages (pure oviparous or viviparous) based on the HPD interval overlapping the q and Q12 expectations for each

category. The proportions of deep viviparous and deep oviparous backcrossed individuals in the population were compared using an exact binomial test. The q-values estimated by ENTROPY were strongly correlated with those from admixture (r=0.99).

Genomic and Geographic Cline Analyses

For geographic cline analysis, a 1-dimensional transect was approximated from the 2-dimensional sampling scheme (Fig. 1). This was achieved by performing a simple linear regression analysis to model the relationship between longitude and latitude coordinates, and the regression line became the transect line. Coordinates for the transect line and individual sample locations were transformed from the WSG84 coordinate system to the Austria Lambert coordinate reference system, using the sf package v1.0.14 (*19*). The closest point on the transect line to each sampled individual was established using the nearest site function. The bearing of the transect line was estimated using the Haversine formula between the coordinates at the start and end points of the transect line, having first converted them to radians. The bearing was converted back into degrees and used to calculate the distance in kilometres between the point on the transect of the individual with the lowest latitude and each other individual position on the transect, resulting in a linear distance for each individual along the transect. Ancestrally informative markers (3506 loci) were selected as those with a Wright's F_{ST} of 0.7 or higher between the two parental lineages. These markers were subset from the imputed genotypes and recoded (0, 0.5, 1). Individuals were grouped into 50 m intervals taken along the transect. Any 50 m group that contained no individuals was dropped and the average allele frequency for each SNP in each group was calculated. The distance of the remaining groups was mean centred and variance standardised.

Geographic and genomic clines analyses was carried out using the bgchm R package v0.0.0.9000 (*20*, *21*). The geographic analysis used the est_geocl function with the hier flag to TRUE, which applies a Bayesian hierarchical model fitting locus-specific allele frequencies against geographic distance on the logit scale; these models are fully described by Gompert et al (*20*). An approximate precision of 0.01 was used along with 10000 iterations and thinning every 5 iterations.

Genomic cline analysis was carried out using est_genocl function and the same 3506 ancestry-informative loci used in the geographic clines. Based on the previously assigned q-values, the parental genotypes (those with ancestry proportion of 95% or greater) were used in the est_p function, with default settings, to estimate the parental allele frequencies. These were passed to the est_genocl function along with the remaining, hybrid, genotypes. The

previously calculated q-values were supplied as the hybrid indexes. The hier flag was used to specify a Bayesian hierarchical logit-logistic model which fits locus-specific ancestry probabilities as a function of hybrid index (50); the model was run for 10000 iterations, thinning every 5.

Trace plots were visually inspected to ensure that the model had converged. Additionally, Rhat values for all the parameters in the model ranged between 0.999 and 1.004 indicating no issues with convergence. Outliers were determined based on the 90% equal-tail probability intervals excluding 1 for the cline gradient, or 0.5 for the cline centre. Cline gradients < 1 are classified as shallow clines, while gradients > 1 are considered steep clines (*20*, *21*). Loci with cline centres below 0.5 are considered to have a credible excess of oviparous ancestry, while cline centre above 0.5 suggests a credible excess of viviparous ancestry.

Genomic cline analysis was additionally carried out for all 508,448 loci, using non-hierarchical models (hier=FALSE). To get starting standard deviation values for the cline centre and cline gradient, five sets of 1000 SNPs were run in hierarchical models, similar to those using the ancestry-informative loci, using the default setting of 4000 iterations. The mean sdc (0.478) and sdv (0.494) across these 5 models were then used in the single locus models, passed to the function using the SDc and SDv flags respectively, all other settings were kept the same as the ancestry-informative loci model. Prior to further analyses sum-to-zero constraints were applied to the cline centres and gradients using the sum2zero function. This approach allows estimates for all loci, which is not computationally feasible using a hierarchical framework.

To calculate F_{IS}, linkage disequilibrium, and admixture value modality along the transect for the full SNP dataset, individuals were split into 10 groups of roughly even size (27 individuals per group; final group n = 23). We divided the transect by samples rather than geographic distance to maximise and even the biological sample size. For each group and using the imputed dataset analysed in R, observed heterozygosity (Ho) was calculated per locus as the proportion of heterozygotes. Unbiased expected heterozygosity (Hs) was computed per locus using Nei's gene-diversity estimator with the standard sample-size correction. F_{IS} was then calculated as $F_{IS} = 1 - (\sum Ho)/(\sum Hs)$ across loci. Confidence intervals (95%) were obtained by bootstrapping individuals within populations (resampling with replacement, preserving population sizes). In addition, for each group, linkage

disequilibrium (r2) between neighbouring SNP pairs was quantified with PLINK using a sliding window of 2, a maximum distance between SNPs of 1Mbp, and the threshold for reported r2 of 0.

Local recombination rate inference

To establish recombination rate across the genome, autosomal markers were lifted over from a previously developed linkage map that was placed on a *Z. vivipara* reference genome from a different phylogeographic lineage (*11*). A chain file between the previous and current genomes was generated using flo v1.1.0 (*22*) and within the blast options the tile size was set to 12, minimum sequence identity to 0.7, and fastMap flag was used. Crossmap v0.7.0 (*23*) was used to lift across the loci from the original linkage map in vcf format. Loci lifted over correctly on to the matching chromosome in the Eastern Oviparous genome were retained along with their genomic position, in cM, from the original map and their physical position, in Mbp, from the Eastern Oviparous genome.

Recombination rates were calculated for each autosome. First, a simple linear regression was carried out, modelling the relationship between the physical and genetic positions. Cook's distance (Di) was calculated for each point and any point with Di greater than 3 times the mean was considered to be an outlier and removed; these more likely represent discordance between genome versions than biologically meaningful recombination points. The chromosome was then further divided into subsections based on the presence of continuous data, with splitting occurring at any region with a gap of 5 Mbp or more. These long gaps of missing data were treated as separate sections and excluded, as were any regions containing 50 or fewer loci. Each subset was modelled using shape constrained additive models with the scam R package v1.2-14 (*24*). A monotonic piecewise interpolated spline was used when a simple regression indicated a positive relationship, otherwise a monotonic piecewise decreasing spline was used. In both cases 25 knots are used to fit the data. A SNP-specific recombination rate, in cM/Mb, was then calculated as the slope of the predicted spline at that location in the genome for each SNP in a region with a spline fitted. Chromosomes 9 and 11 were excluded from further analysis as they lifted over very poorly. The average recombination rate for each chromosome was calculated. In total, 15087 autosomal SNPs were successfully lifted over and 14899 were mapped onto the expected chromosome. Splines were fitted to 25 separate regions containing between 54 and 1215 loci. An additional 5 regions, having a total of 114 SNPs, were dropped because of containing a low number of loci.

To evaluate a relationship between genomic cline steepness and recombination rates, simple linear regressions were conducted on both the SNP specific values and the chromosome averages.

Supplementary Figures

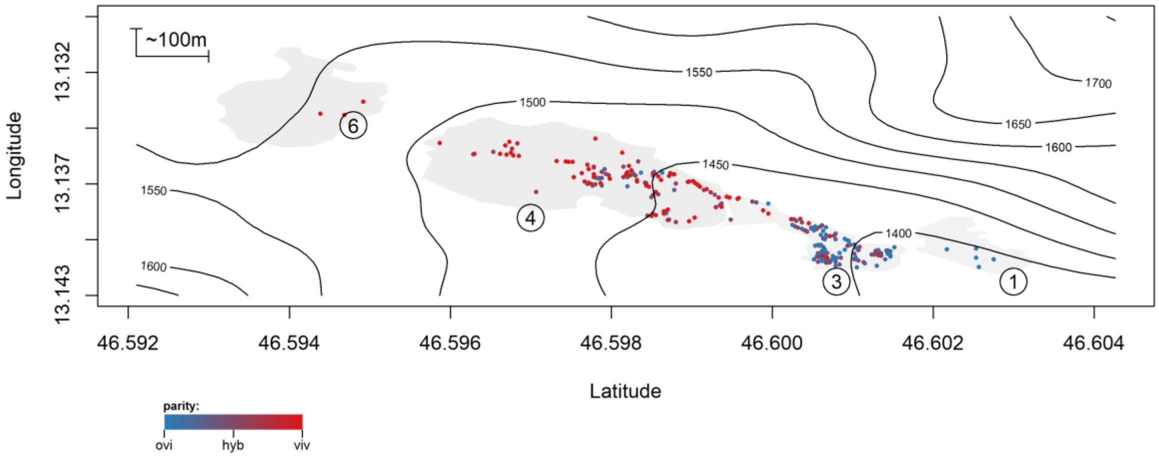

Supplementary Figure 1. Location of individuals (oviparous, hybrid and viviparous in blue, purple, red) included in the study, with parity mode inferred from genomic analysis. Numbers in circles refer to sampling meadows (sites 1,3,4, and 6 included from ref. [22]) and grey areas indicate suitable habitat at the study site.

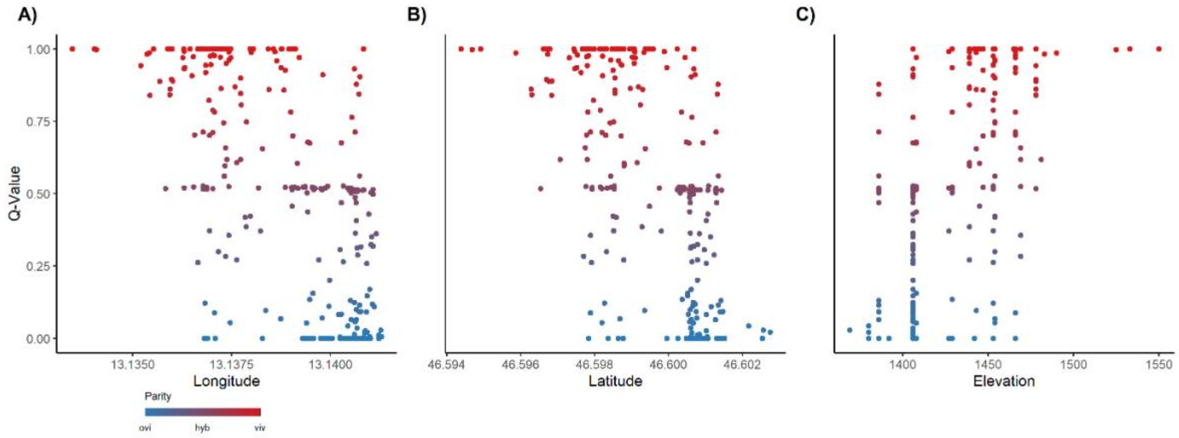

Supplementary Figure 2. Q values (point estimates) plotted against longitude, latitude, and elevation (masl) for all individuals at the hybrid zone.

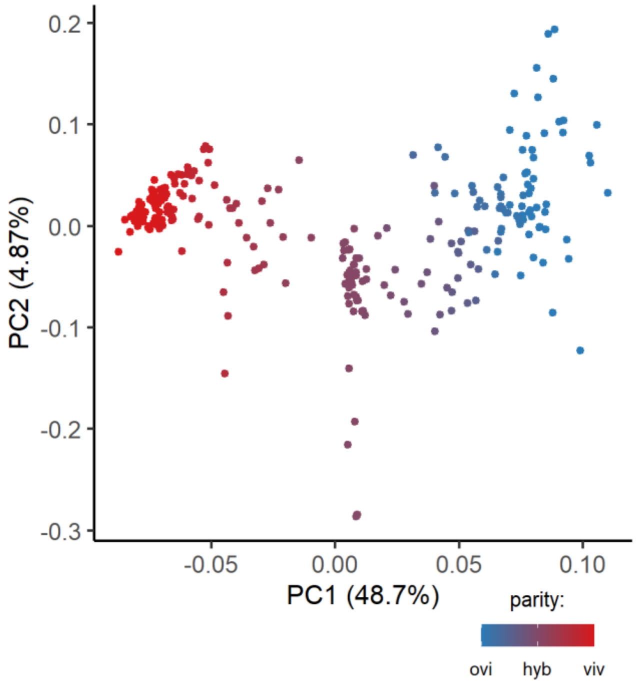

Supplementary Figure 3. Principal components analysis displaying each individual lizard along PC1 vs PC2, coloured by ancestry proportion q-value for parity mode as inferred from 508,448 SNPs (266 individuals).

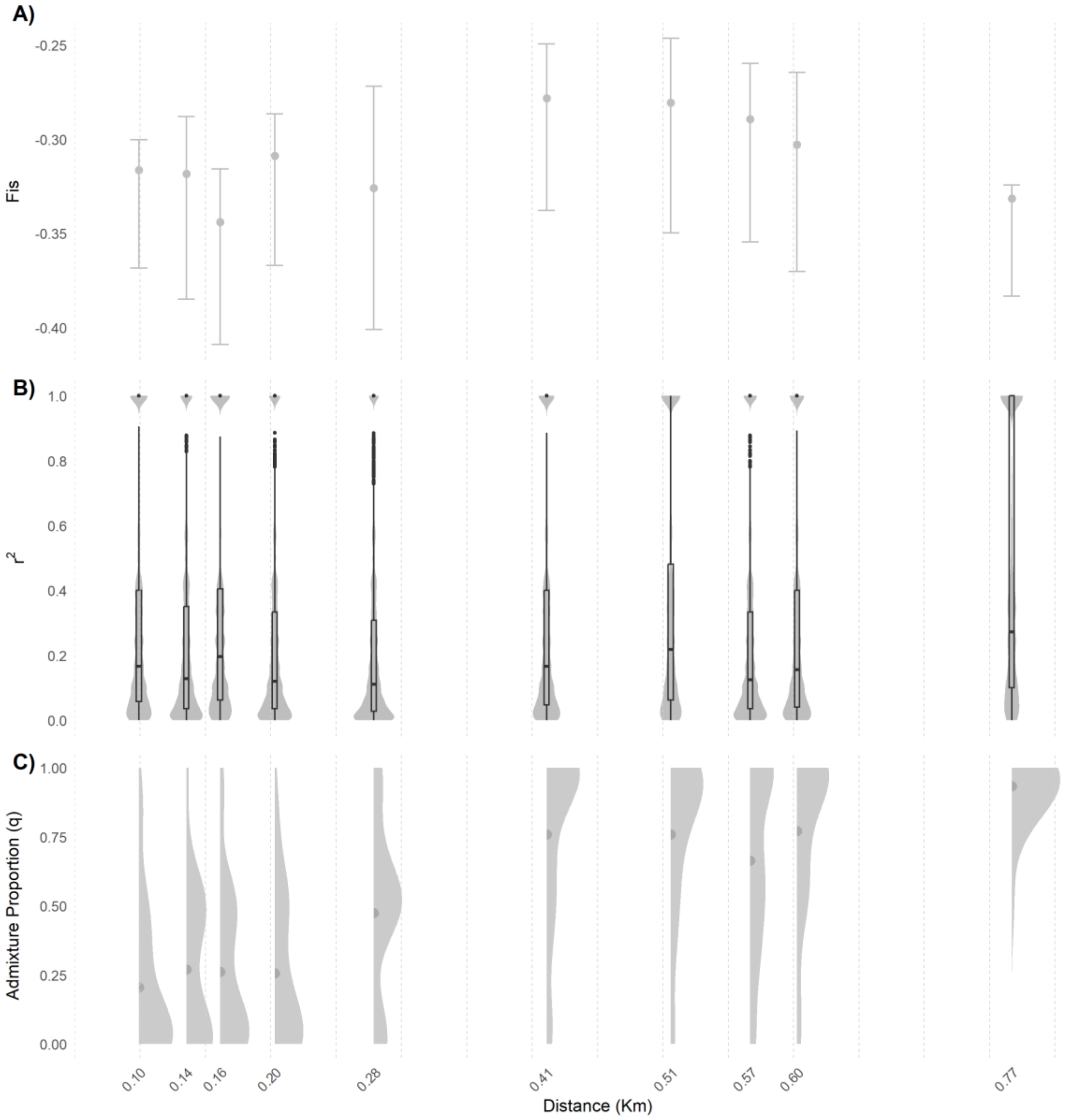

Supplementary Figure 4. Population-level linkage disequilibrium and inbreeding within each grouping along the spatial transect (0 km is the lower elevation, oviparous-dominated) and ordered by midpoint distance (km). (A) Population-wide inbreeding coefficient (F_{IS}) with 95% bootstrap confidence intervals plotted at each group. (B) Distribution of pairwise LD (r^2) for neighbouring SNP pairs within each group, shown as violins (truncated at 0 and 1) with overlaid boxplots (horizontal line = median). (C) Distribution of admixture proportions (q-values) within each grouping along the transect, dark grey semi-circles show the mean ancestry proportion of that group.

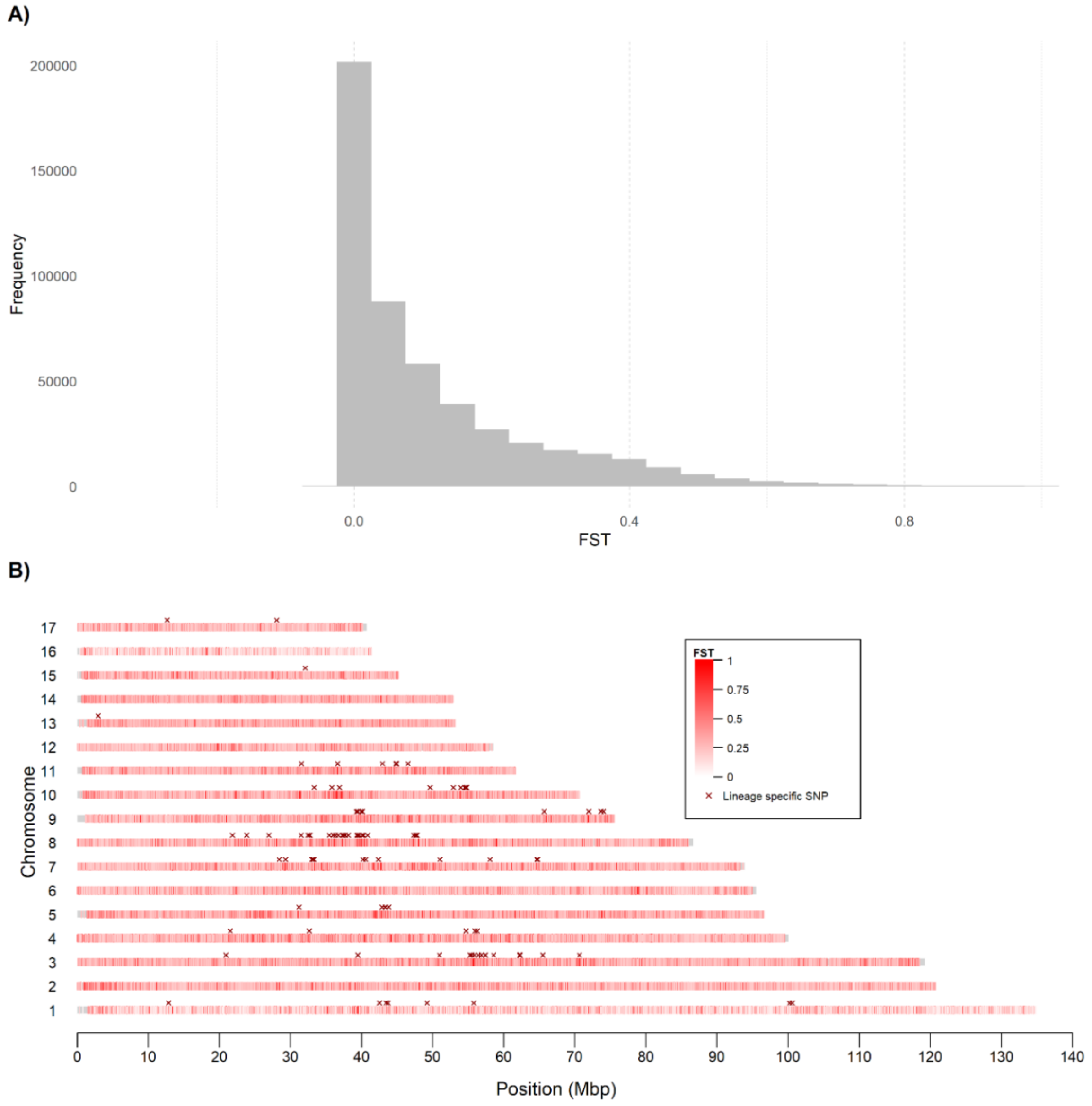

Supplementary Figure 5. (A) The frequency of SNP-specific F_{ST} across all 17 autosomal chromosomes. (B) The location of these F_{ST} values placed along each chromosome and their value reflected in red-scale (see legend). Locations of SNPs that were identified as being fixed between the two parity mode lineages are marked with red crosses.

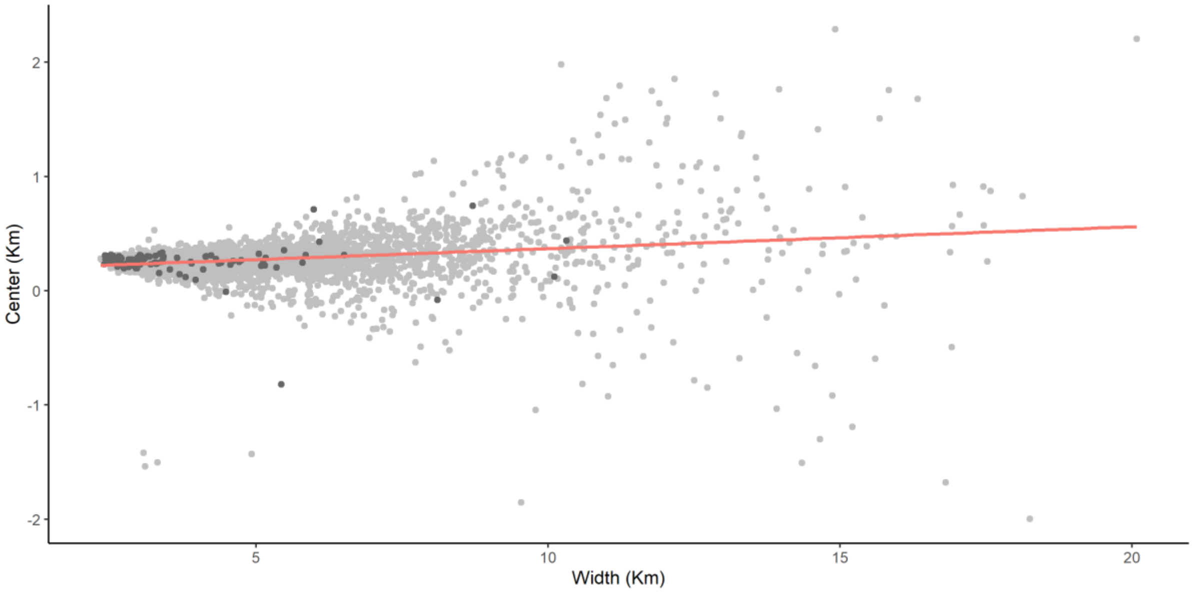

Supplementary Figure 6. Dotplot of geographic cline centres vs cline widths for 3506 ancestrally-informative SNPs in light grey and correlation line in red (slope = 0.019, SE = 0.002, $p < 0.001$). The 107 SNPs fixed between lineages are shown in dark grey.

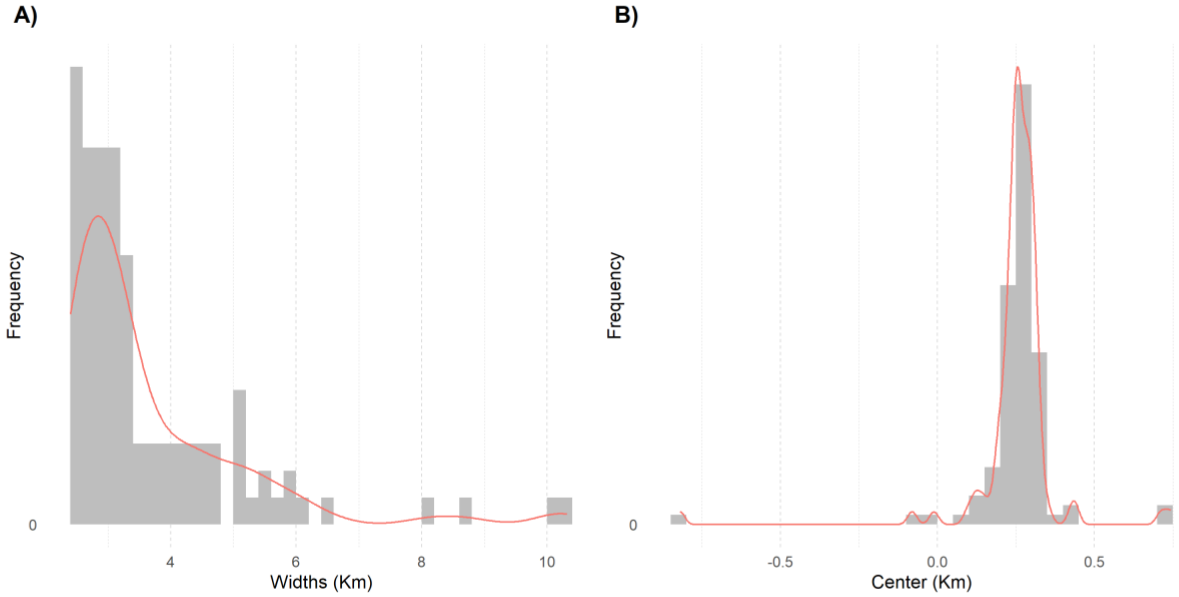

Supplementary Figure 7. (A) Frequency of the geographic cline widths and (B) cline centres for the 107 SNPs that are fixed between parity mode lineages, along with a smoothed approximation of the distribution shape, in red.

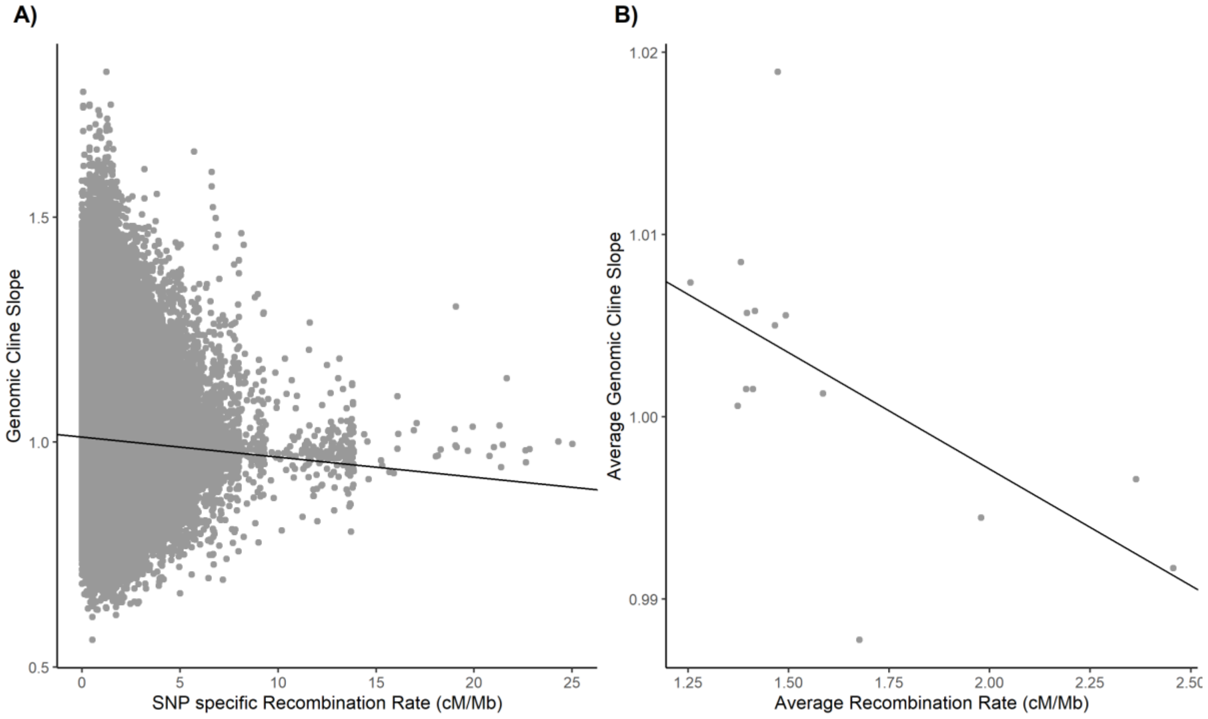

Supplementary Figure 8: Relationship between steepness of the genomic cline slope and recombination rate at individual loci (A) and chromosome level (B).

Supplementary Results:

Genomic cline for all loci

Across the 508448 loci a total of 97331 loci deviated from the null expectations, based on genome average admixture (Supplementary Table 7). Of these 2781 had a credibly steep cline and 827 had a credibly shallow cline. When considering cline centres, 49935 of the loci had values indicating excess oviparous ancestry while 45528 had cline centres indicating excess viviparous ancestry.

There were only 1740 loci that were outliers for both the cline centres and cline widths (Supplementary Table 7). As with the ancestrally informative markers, the majority of loci with credibly shallow or steep genomic clines are neutral for excess ancestry. However, 7.73% of the shallow clines display excess oviparous ancestry and 17.17% display excess viviparous ancestry indicating positions in the genome with reduced barriers to gene flow biased to alleles from each of these lineages respectively. Similarly, 38.367% of steep clines display excess oviparous ancestry and 16.793% display excess viviparous ancestry suggesting lineage-specific barriers to introgression. All four types of outlier were distributed across the genome and were found on every autosome (Supplementary Table 8).

Supplementary tables:

Supplementary Table 1 (in excel sheet). Sample information for each individual included in the study: ID number, geographic coordinates (longitude east, latitude north, altitude m), admixture q-proportion to viviparous ancestry, assigned category based on genomic background (oviparous, viviparous, hybrid), accession number, and sequencing coverage.

Supplementary Table 2 (in excel sheet). Position (chromosome, site) and F_{ST}, of ancestry-informative and fixed SNPs in our analyses.

Supplementary Table 3 (in excel sheet). Per individual q- and Q12-values (with HPDs) as inferred from Entropy analysis and used to assign individuals to hybrid category.

Supplementary Table 4. Number of each type of outlier, from the genomic cline analysis on ancestrally informative markers, that were located on each chromosome. The percentage out of all the loci analysed on each chromosome is also shown.

Chromosome	Shallow Gradient	Steep Gradient	Centre Low (Ovi Anc)	Centre High (Viv Anc)	Neutral SNPs	Total SNPs
1	11 (4%)	47 (17%)	19 (6.9%)	11 (4%)	201 (72.6%)	277
2	10 (5.8%)	27 (15.8%)	16 (9.4%)	2 (1.2%)	123 (71.9%)	171
3	31 (8.7%)	21 (5.9%)	16 (4.5%)	24 (6.8%)	269 (75.8%)	355
4	32 (9.6%)	41 (12.2%)	13 (3.9%)	9 (2.7%)	244 (72.8%)	335
5	28 (7%)	17 (4.3%)	24 (6%)	12 (3%)	321 (80.7%)	398
6	33 (24.1%)	2 (1.5%)	2 (1.5%)	5 (3.6%)	97 (70.8%)	137
7	63 (13.7%)	25 (5.4%)	16 (3.5%)	12 (2.6%)	349 (76%)	459
8	22 (6.5%)	59 (17.4%)	10 (2.9%)	9 (2.6%)	245 (72.1%)	340
9	22 (8.2%)	43 (16%)	23 (8.6%)	15 (5.6%)	175 (65.3%)	268
10	7 (3.6%)	26 (13.5%)	12 (6.2%)	7 (3.6%)	141 (73.4%)	192
11	12 (6.6%)	14 (7.7%)	10 (5.5%)	7 (3.8%)	143 (78.1%)	183
12	2 (2.2%)	10 (11%)	2 (2.2%)	9 (9.9%)	69 (75.8%)	91
13	6 (7.9%)	2 (2.6%)	8 (10.5%)	7 (9.2%)	54 (71.1%)	76
14	2 (5.9%)	3 (8.8%)	4 (11.8%)	1 (2.9%)	25 (73.5%)	34
15	16 (17.4%)	4 (4.3%)	8 (8.7%)	9 (9.8%)	58 (63%)	92
16	10 (16.4%)	0 (0%)	8 (13.1%)	3 (4.9%)	40 (65.6%)	61
17	5 (13.5%)	1 (2.7%)	4 (10.8%)	3 (8.1%)	26 (70.3%)	37

Supplementary Table 5. Relationship between each type of outlier from the genomic cline analysis on ancestrally informative markers.

	Centre			
Slope	Low (Ovi Anc)	High (Viv Anc)	Neutral	Total
Shallow	1	9	302	312
Steep	41	17	284	342
Neutral	153	119	2580	2852
Total	195	145	3166	3506

Supplementary Table 6. Probabilities of cline shape for each chromosome and genome wide, based on taking 10000 samples for the posterior distribution from the ancestrally informative, hierarchical mode.

Chromosome	Probability of steep cline	Probability of viviparous ancestry
1	0.8306	0.3473
2	0.7534	0.3071
3	0.7561	0.4921
4	0.758	0.3823
5	0.7506	0.3946
6	0.5947	0.4724
7	0.6773	0.4276
8	0.7933	0.4248
9	0.7391	0.4439
10	0.7982	0.4574
11	0.7593	0.5049
12	0.7472	0.492
13	0.7216	0.5335
14	0.7487	0.3396
15	0.566	0.4591
16	0.6342	0.3821
17	0.6243	0.3988
Genome Wide:	0.7420	0.4256

Supplementary Table 7. Relationship between each type of outlier from the genomic cline analysis on all 508448 markers.

	Centre			
**Slope**	Low (Ovi Anc)	High (Viv Anc)	Neutral	**Total**
Steep	1067	467	1247	2781
Shallow	64	142	621	827
Neutral	48804	44919	411117	504840
**Total**	49935	45528	412985	508448

Supplementary Table 8. Number of each type of outlier, from the genomic cline analysis on all 508448 markers, located on each chromosome. The percentage out of all the loci analysed on each chromosome is also shown.

Chromosome	Shallow Gradient	Steep Gradient	Centre Low (Ovi Anc)	Centre High (Viv Anc)	Neutral SNPs	Total SNPs
1	60 (0.1%)	498 (1%)	5524 (11.5%)	4826 (10.1%)	37427 (77.9%)	48016
2	42 (0.1%)	318 (0.7%)	4951 (11.3%)	2747 (6.3%)	35963 (82.1%)	43814
3	96 (0.2%)	217 (0.5%)	3986 (9.1%)	4306 (9.8%)	35485 (80.7%)	43963
4	43 (0.1%)	262 (0.7%)	4571 (11.5%)	2977 (7.5%)	31929 (80.5%)	39648
5	69 (0.2%)	201 (0.6%)	3799 (10.7%)	3521 (9.9%)	28020 (78.9%)	35515
6	104 (0.3%)	80 (0.2%)	3159 (9.2%)	3228 (9.4%)	27695 (81%)	34192
7	95 (0.3%)	129 (0.4%)	4192 (11.5%)	3057 (8.4%)	28930 (79.7%)	36313
8	63 (0.2%)	289 (0.9%)	3102 (9.5%)	3253 (10%)	26080 (79.8%)	32662
9	20 (0.1%)	301 (1.1%)	2101 (7.5%)	3188 (11.4%)	22517 (80.6%)	27936
10	41 (0.2%)	149 (0.6%)	2399 (9.3%)	2183 (8.4%)	21219 (81.9%)	25900
11	46 (0.2%)	98 (0.4%)	2200 (8.6%)	2546 (10%)	20742 (81.2%)	25544
12	34 (0.1%)	86 (0.4%)	1623 (7%)	2296 (9.9%)	19285 (82.9%)	23270
13	22 (0.1%)	52 (0.2%)	1673 (8%)	2014 (9.6%)	17202 (82.2%)	20916
14	10 (0%)	42 (0.2%)	1804 (8.7%)	1331 (6.4%)	17617 (84.8%)	20772
15	38 (0.2%)	16 (0.1%)	1958 (10.8%)	1485 (8.2%)	14626 (80.8%)	18108
16	31 (0.2%)	21 (0.1%)	1599 (10%)	1373 (8.6%)	13058 (81.3%)	16057
17	13 (0.1%)	22 (0.1%)	1294 (8.2%)	1197 (7.6%)	13322 (84.2%)	15822

Supplementary Table 9. Chromosome average recombination rates in cM/Mb for all autosomes. Chromosomes 9 and 11 are excluded (NA) due to poor coordinate conversion between genome assemblies.

Chromosome	Average Recombination Rate (cM/Mb)
1	1.473
2	1.382
3	1.412
4	1.256
5	1.466
6	2.364
7	1.374
8	1.397
9	NA
10	1.586
11	NA
12	1.417
13	1.395
14	1.493
15	1.676
16	1.979
17	2.457

Supplementary References

1. Recknagel H, Carruthers M, Yurchenko AA et al. The functional genetic architecture of egg-laying and live-bearing reproduction in common lizards. Nature Ecology and Evolution. 2021;5:1546-1556.
2. McLennan D, Recknagel H, Elmer KR, Monaghan P. Distinct telomere differences within a reproductively bimodal common lizard (*Zootoca vivipara*) population. Functional Ecology. 2019;33:1917-1927.
3. Recknagel H, Elmer KR. Differential reproductive investment in co-occurring oviparous and viviparous common lizards (*Zootoca vivipara*) and implications for life-history trade-offs with viviparity. Oecologia. 2019;190:85-98.
4. Jauslin R, Tillé Y. WaveSampling: Weakly associated vectors (WAVE) sampling (manual). 2022
5. R Core Team. R: A language and environment for statistical computing (manual). 2021.
6. Wingett SW, Andrews S. FastQ Screen: A tool for multi-genome mapping and quality control. F1000Res. 2018;7:1338.
7. Wood DE, Lu J, Langmead B. Improved metagenomic analysis with Kraken 2. Genome Biol. 2019;20:257.
8. Bolger AM, Lohse M, Usadel B. Trimmomatic: a flexible trimmer for Illumina sequence data. Bioinformatics. 2014;30:2114-2120.
9. Xie H, Recknagel H, Li C et al. Genome-wide barriers to gene flow reveal the genetic basis of viviparity evolution in a lizard. Current Biology. 2026;36:1-14.
10. Vasimuddin M, Misra S, Li H, Aluru S. Efficient architecture-aware acceleration of BWA-MEM for multicore systems. 2019;2019 IEEE international parallel and distributed processing symposium:314-324.
11. Yurchenko AA, Recknagel H, Elmer KR. Chromosome-Level Assembly of the Common Lizard (*Zootoca vivipara*) Genome. Genome Biol Evol. 2020;12:1953-1960.
12. Danecek P, Bonfield JK, Liddle J et al. Twelve years of SAMtools and BCFtools. Gigascience. 2021;10:giab008.
13. Okonechnikov K, Conesa A, García-Alcalde F. Qualimap 2: advanced multi-sample quality control for high-throughput sequencing data. Bioinformatics. 2016;32:292-294.
14. Purcell S, Neale B, Todd-Brown K et al. PLINK: a tool set for whole-genome association and population-based linkage analyses. Am J Hum Genet. 2007;81:559-575.
15. Browning BL, Tian X, Zhou Y, Browning SR. Fast two-stage phasing of large-scale sequence data. Am J Hum Genet. 2021;108:1880-1890.
16. Alexander DH, Novembre J, Lange K. Fast model-based estimation of ancestry in unrelated individuals. Genome Res. 2009;19:1655-1664.
17. Goudet J, Jombart T. Hierfstat: Estimation and tests of hierarchical f-statistics. R package version 05-11. r-project.org
18. Shastry V, Adams PE, Lindtke D et al. Model-based genotype and ancestry estimation for potential hybrids with mixed-ploidy. Mol Ecol Resour. 2021;21:1434-1451.
19. Pebesma E, Bivand R. Spatial data science: With applications in R. taylorfranciscom. 2023
20. Gompert Z, DeRaad DA, Alex Buerkle C. A next generation of hierarchical Bayesian analyses of hybrid zones enables direct quantification of variation in introgression in R. Ecology and Evolution. 2024;14:e70548.
21. Gompert Z. bgchm: Bayesian analyses of hybrid zones with hamiltonian monte carlo (manual). 2025

22. Pracana R, Priyam A, Levantis I, Nichols RA, Wurm Y. The fire ant social chromosome supergene variant Sb shows low diversity but high divergence from SB. Mol Ecol. 2017;26:2864-2879.
23. Zhao H, Sun Z, Wang J, Huang H, Kocher JP, Wang L. CrossMap: a versatile tool for coordinate conversion between genome assemblies. Bioinformatics. 2014;30:1006-1007.
24. Pya N. scram r Package: Shape Constrained Additive Models. R package documentation. 2023
